# Tailoring Lithium Fluoride Interface for Dendrite-Free Lithium Anode to Prolong the Cyclic Stability of Lithium–Sulfur Pouch Cells

**DOI:** 10.1186/s11671-022-03745-w

**Published:** 2022-11-23

**Authors:** Li Zhang, Yu Jiao, Fan Wang, Mingjie Zhou, Yin Hu, Yichao Yan, Fei Li, Tianyu Lei, Bo Chen, Wei Chen

**Affiliations:** 1 State Key Laboratory of Electronic Thin Films and Integrated Devices, University of Electronic Science and Technology of China, Chengdu, 610054 China; 2 Tianfu Co-Innovation Center, University of Electronic Science and Technology of China, Chengdu, 610213 China; 3 College of Science, Xichang University, Xichang, 615000 China; 4 Institute of Microelectronics of Chinese Academy of Sciences, Beijing, 100029 China

**Keywords:** Solid electrolyte interface, Lithium dendrites, Lithium–sulfur pouch cells, Commercialization

## Abstract

Lithium–sulfur (Li–S) cells have been regarded as attractive alternatives to achieve higher energy densities because of their theoretical specific energy far beyond the lithium-ion cells. However, the achieved results of Li–S cells are exaggerating the cycle performance in their pouch formats because the considerable works are based on the coin cells where flood electrolyte and endless Li supply ensure the Li metal with nature structure features, resulting in a negligible effect on cycle performance caused by the Li dendrites and electrolyte dissipation during cycles. Herein, we demonstrate a strategy to enable the Li metal with lithium fluoride (LiF)-rich solid electrolyte interface via integrating a reinforced interface (RI) embedded with nano-LiF particles on the surface of the Li metal anode. The RI interface enables the solvent molecules of the electrolyte to gain fewer electrons from Li anode, resulting in a lower leakage current of assembled RI||Li–S cell (~ 0 μA) than pristine Li anode (~ 1.15 µA). Moreover, these results show that suppressing lithium dendrite growth is more urgent than inhibiting the shuttle effect of polysulfides in the pouch cell format. As a result, the RI layer-engineered Li metal bears witness to the cyclic stability of Li anode over 800 h, thus achieving stable cycles of Ah-scale Li–S pouch cell with an energy density of 410 Wh/kg at a current of 200 mA per cell. Our study demonstrates that the suppression of lithium dendrites by the RI could be a promising method to prolong the cycle number of Li–S pouch cells.

**Supplementary Information:**

The online version contains supplementary material available at 10.1186/s11671-022-03745-w.

## Introduction

Lithium–sulfur (Li–S) cells have been adopted as the next-generation storage device because of their potential in ultra-high specific energy density and cost-effective of sulfur [1–5]. The common vision of all researchers is to move the Li–S cells from laboratory-scale coin formats to industrial-scale pouch cells [6–8]. In addition, Li–S batteries have been demonstrated with higher safety than the existing lithium-ion cells in the case of short circuit and nail test due to their special redox progresses in which no hazardous oxygen radical was released as suffering thermal runaway [9]. However, the shuttle effect of long-chain polysulfides (Li_2_S_*n*_; 3 ≤ *n* ≤ 8) [10–13] and the growth of lithium dendrites [7, 14–16] on the surface of lithium metal anode make the cyclic stability of Li–S cells much lower than the market demand (80% capacity retention after 600 cycles) [17]. Although considerable effects have been made to stabilize the cycle stability of Li–S cells to more than 1000 cycles via designing polar adsorption materials [18], catalytic materials [19, 20], multifunctional separator [21] or other technologies [22], the reported methods are based on the laboratory-scale coin cells where the volume ratio of electrolyte to the loading of sulfur (*E*/*S*) is usually higher than 30 μL/mg and the capacity ratio of lithium metal anode to the sulfur cathode (*N*/*P*) is higher than 45 [23]. High *E*/*S* and *N*/*P* ratios make the obtained electrochemical performances based on coin cells generally overestimated, resulting in the poor cycling stability of commercially visualized pouch cells with the same strategy (< 60 cycles) [17, 24].

To date, the improvements in cycle performance of Li–S pouch cells are usually focused on the suppression of the shuttling effect, as it is widely believed that the loss of polysulfides from the cathode is the main reason for the degradation of cyclic stability of Li–S cells based on their working principle [6, 25]. Indeed, the long-chain polysulfides (Li_2_S_n_) generated by the S_8_ in the discharge process are easily dissolved in the ether electrolyte, thereby leaving the shuttling in the electrolyte, thus resulting in the decrease in capacity, Coulomb efficiency and cycle stability of Li–S cells [26]. In the pouch cell, the dosage of the injected electrolyte usually needs to be carefully calculated to ensure the overall energy density of assembled cell is comparable to the existing lithium-ion cells [27–29]. Accordingly, a low dosage of electrolyte with *E*/*S* < 5 is needed. However, the low-dose electrolytes in the pouch cell formats result in limited solubility of polysulfides in the electrolyte, thereby limiting the shuttle effect of polysulfides in pouch cells [30, 31]. In other words, the main reason that influences the stable of Li–S pouch cells may not be the dissolution of polysulfides. By contrast, the growth of lithium dendrites caused by the random deposition of lithium ions and the mechanical fragility of solid electrolyte interphase (SEI) on the surface of lithium metal gives rise to the broken and repeated reconstruction of SEI, allowing the emergence of “dry” electrode interface and the increase in internal concentration polarization [7, 32, 33].

Inhibiting lithium dendrites is one of the most effective ways to commercialize the Li–S pouch cells. Although some efforts such as adding electrolyte additive [28, 34] or preparing a coating layer [23, 35] can improve the stability of Li–S pouch cells, the corresponding strategies are extremely difficult to apply to the existing industrial processes due to their complex synthetic processes or high cost. Moreover, the use of dry room (RH = ~ 1.55%) during cell assembly surely results in the growth of a LiOH layer on the surface of Li metal anode due to the H_2_O corrosion, which makes the existing strategies about their potential on suppressing the lithium dendrites inevitably challenged. Herein, we demonstrate a simple approach to uniformly cast a reinforced interface (RI) embedded with nano-LiF particles on the surface of Li metal anode. This strategy not only adapts to the existing industrial process assembly, but also allows the modified Li with fewer parasitic side reactions (~ 0 µA leak current) and stable striping/plating performance (more than 800 h). When paired with S cathode, the assembled Ah-level Li–S pouch cells with the RI layer-engineered Li metal (RI||Li) anode show a high energy density of more than 410 Wh/kg and deliver stable cycle stability even the *E*/*S* ratio = 4 and *N*/*P* ratio = 2. Our formulation of RI layer on the Li surface satisfies the critical requirements for the practical yield and application of Li metal.

## Experimental Section

### Methods and Chemicals

Lithium fluoride (LiF), styrenic block copolymers (SBC), tetrahydrofuran (THF) and 1-methyl-2-pyrrolidinone (NMP) were commercially purchased from Aladdin without further purification. The THF was stored in an Ar-protected glove box before use. 4 Å molecular sieve was employed to adsorb the water molecules in the THF solution. Sulfur (S), acetylene black (AB), polyvinylidene fluoride (PVDF), lithium bis(trifluoromethane sulfonimide) (LiTFSI), 1,2-dimethoxyethane (DME) and Li-S electrolyte (1 M LiTFSI in a mixture of 1,3-dioxolane (DOL) and DME (1:1 v/v) with 2 wt% lithium nitrate (LiNO_3_) as additive were purchased from DoDoChem Co., Ltd. Lithium (Li) metal anode (~ 450 µm) applied for coin cells and Li tape with the thickness of 100 µm applied for Li–S pouch cells were purchased from China energy lithium Co., Ltd.

### Fabrication of RI||Li and Characterization

For the fabrication of RI||Li, SBC powder was dissolved into THF solution with a concentration of 200 mg mL^−1^. Then, the LiF nanoparticles were added to the above solution to form a uniform RI solution. The RI||Li was obtained by gradually solidifying the RI layer at 30 °C for 2 h and then 60 °C for 24 h to vaporize the free THF solvent (Additional file 1: Fig. S1). The morphologies of Li metal and Li deposits were observed via scanning electron microscope (SEM) (FEI, NANOSEI 450).

### Electrochemical Measurement

Before test, the Li anode was stored in different conditions. For instance, the pristine Li and RI||Li were exposed in dry room (RH = 1.55%, temperature ~ 25 °C) with scheduled 7 days for the case of investigating the environmental tolerance of dry room. For the symmetrical Li||Li manufacturing, the coin cells (CR-2032 or 2025) were adopted to assemble the symmetrical cell. Celgard 2400 polypropylene (PP) was used as the separator. The electrochemical performance was monitored using a cell cycler (CT200 A, Wuhan LAND Electronic Co., Ltd). Electrochemical impedance spectroscopy (EIS) was performed on CHI660E or CHI604E electrochemical workstation (Shanghai Chenhua Instrument Co., Ltd).

For the preparation of S electrode applied for coin cell, typically, commercial AB, S powder, and PVDF powder with a mass ratio of 3:6:1 were firstly mixed in NMP solvent, stirred for 24 h under the protection of Ar atmosphere, and then were uniformly cast on the surface of carbon coated aluminum foil. For pouch cell tests, a similar method was used for the slurry except the electrode was double-coated. The average loading of S for coin cell tests is ~ 1 mg cm^−2^, and for pouch cell is ~ 5 mg cm^−2^ (single side). The amounts of the electrolyte in Li symmetric cells, Li–S coin cells, and Li–S pouch cells are 30 µL/cell, 30 µL/mg, and 4 µL/mg, respectively.

## Results and Discussion

### Design Concept and Hydrophobic Properties of RI||Li

**Fig. 1:**
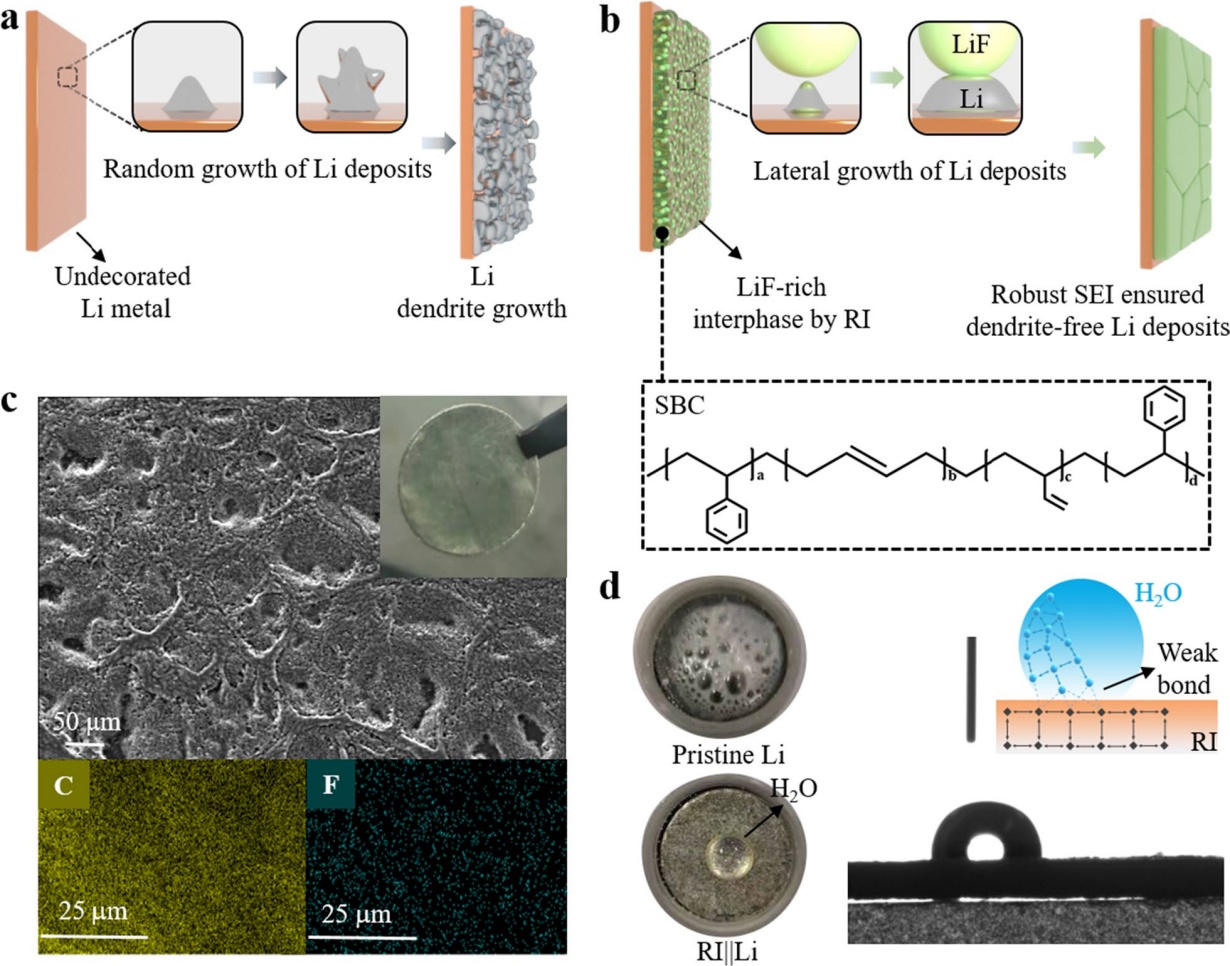
**a** Schematic diagram of Li deposition on the surface of undecorated Li and **b** RI||Li, the molecular structure of SBC is listed at the bottom of plane **b**. **c** SEM image of RI||Li and corresponding element mapping of C and F, the optical image of RI||Li is inserted. **d** Optical images of pristine Li and RI||Li poured by H_2_O droplet, the left is corresponding contact angel and corresponding schematic diagram

LiF is an ideal chemistry to conduce the lateral growth of Li nucleus (Fig. 1a). Therefore, the introduction of LiF nanoparticles into the surface of Li metal can effectively inhibit the Li dendrite growth (Fig. 1b). It inspires us to adopt LiF-rich SEI to decrease the growth of Li dendrites. Besides that, the Li metal to carry out the risk of water corrosion is also needed because the lithium hydroxide (LiOH) layer produced by water corrosion will inevitably passivate the surface of Li metal [36], resulting in the chaotic deposition of Li ions and aggravating growth of Li dendrites. However, the existing dry room (relative humidity (RH) ~ 1.55%) used to assemble pouch cell cannot completely eliminate the existence of H_2_O, so Li metal needs to be appropriately waterproofed to avoid the water corrosion. Therefore, the RI||Li was meticulously designed via uniformly casting the precursors of RI solution containing dispersed LiF nanoparticles and styrenic block copolymers (SBC) on the surface of the lithium metal anode. As shown in Fig. 1b, the hydrophobic polymer material SBC coupling with LiF nanoparticle on the surface of Li metal is hoped to force the lateral growth of Li deposits. Figure 1b also shows the molecular structure of SBC, no ether-based and ester-base groups can be observed in the molecular structure, showing the weak lithium-ion conductivity of SBC. However, the SBC film after aggregation has a large amount of microporous structure, which can be infiltrated by the electrolyte efficiently. The abundant electrolyte transmission channels promote free shuttling of lithium ions in RI layer. As shown in the scanning electron microscopy (SEM) in Fig. 1c, the RI layer is quite uniform and porous, which facilitates the penetration of electrolytes. The corresponding energy spectrum analysis (F element as an indicator of LiF) also confirms the even disperse of LiF on the surface of lithium metal. Moreover, as shown in Fig. 1d, the RI||Li exhibits excellent hydrophobic properties without any violent reaction after dropping water due to the introduction of the SBS layer on the lithium metal surface. In addition, a contact angle of ~ 90° is observed, clearly showing that the water molecules are extremely difficult to enter the RI layer to corrode the lithium metal, thus this performance will greatly facilitate battery assembly. The contact angle of Li–S electrolyte on Li metal electrode and RI||Li electrode are about ~ 16.6° and ~ 15.2°, respectively, suggesting the ultrafast electrolyte wetting capability of RI||Li electrode (Additional file 1: Fig. S2).

### Analysis of RI||Li for Li Platting/Stripping

**Fig. 2:**
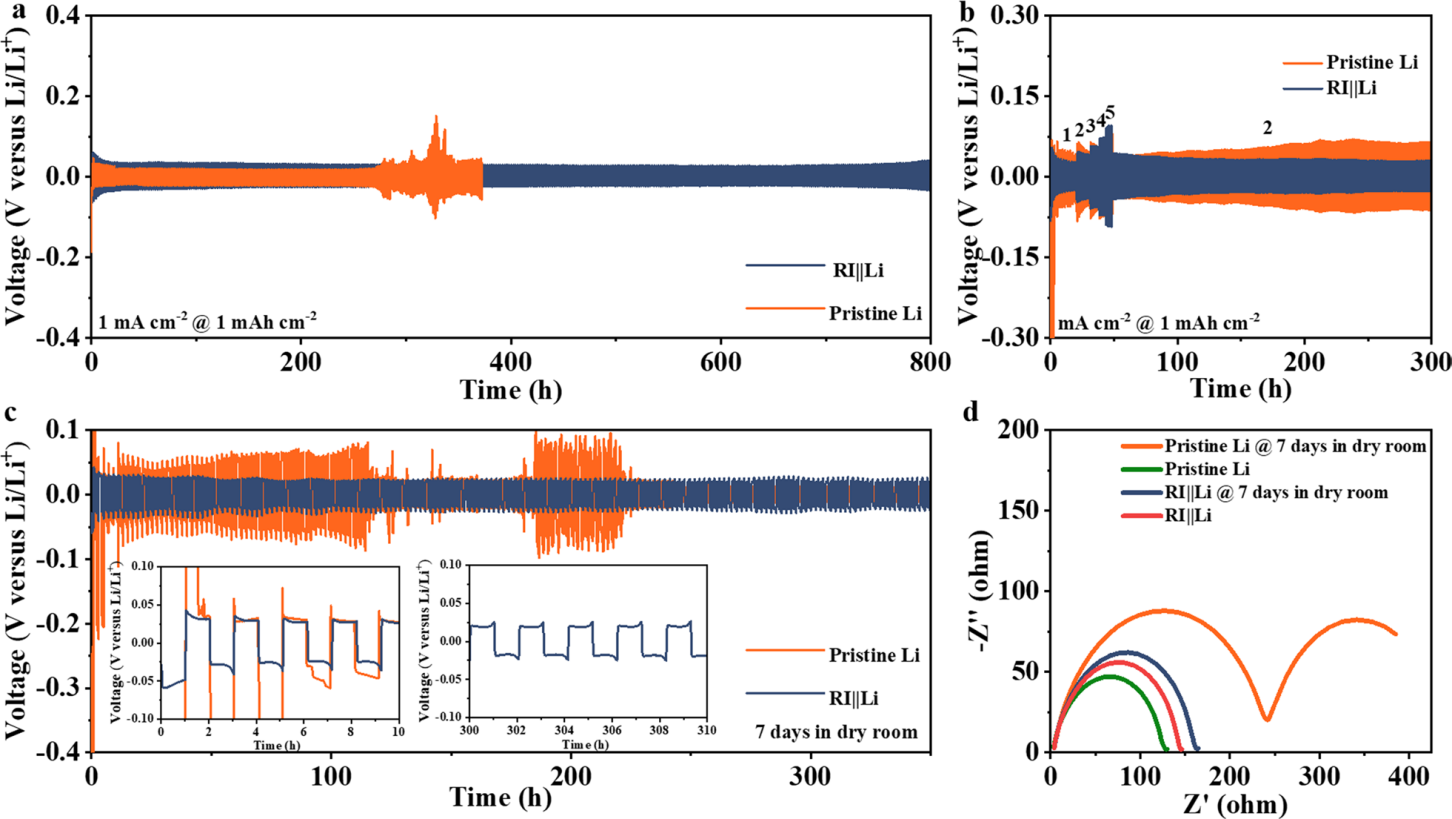
Galvanostatic Li plating/stripping voltage profiles for the RI||Li and pristine Li symmetric cells at a capacity of 1 mAh cm^−2^ with a current density of **a** 1 mAh cm^−2^ and **b** various current densities. **c** Galvanostatic Li plating/stripping voltage profiles for the RI||Li and pristine Li symmetric cells at a capacity of 1 mAh cm^−2^ with a current density of 1 mAh cm^−2^ after stationary in the dry room for 7 days. **d**, EIS results before and after the Li was stored in the dry room for 7 days

The interface stability of Li metal anode is checked via Li symmetrical tests as shown in Fig. 2a, quantitative Li was platted/stripped at a current density of 1 mA cm^−2^ with a capacity of 1 mAh cm^−2^. Obviously, the pristine Li delivers stable cycles before 150 cycles (300 h) since the Li interface maintains stable, while the overpotential turns unstable seriously once cycling exceeds 300 h. This indicates that a large amount of “dead” Li was generated on the surface of lithium metal, which cause the increase in diffusion energy barrier of lithium ion within the electrolyte and the polarization of interface, eventually leading to the rapid depletion of electrolyte. By contrast, Li platting/stripping with stabilized overpotential can be performed more than 800 h with a polarized voltage of ~ 20 mV using the RI||Li electrodes, which means that there is almost no “dead” Li growth to increase the Li ions diffusion energy barrier. In addition, from the zoomed-in voltage profiles inset Fig. 2a, the polarized voltage values of RI||Li electrode are always stable at about 20 mV and no short-circuit can be observed. (Additional file 1: Fig. S3) Further, we tested the rate performance of Li symmetry cells at different lithium plating current densities with a capacity of 1 mAh cm^−2^ to further demonstrate the advantages of RI||Li. As shown in Fig. 2 b, the RI||Li shows stable voltage of ~ 28 mV, 42 mV, 57 mV, 73 mV, and 96 mV at a current density of 1, 2, 3, 4, 5 mA cm ^−2^ with a capacity of 1 mAh cm^−2^, respectively, implying the even flow of lithium ions at the Li interface even as high current density. While, the voltage of pristine Li at a current density of 1–5 mA cm^−2^ with a capacity of 1 mAh cm^−2^ are ~ 44.6 mV, 53.9 mV, 57.7 mV, 69.8 mV, and 89.9 mV, respectively.

Importantly, as shown in Fig. 2c, the Li metal was stored in dry room for 7 days to further confirm the practicability of RI||Li when assembled in the pouch cell format. For the pristine Li, the surface was corroded by water molecules and a LiOH layer was formed. Therefore, an overpotential of ~ 100 mV is needed to ensure the reduction in Li ions on the surface of Li metal during the initial cycles, as shown in the partial enlarged image of Fig. 2c. After eliminating the adverse impact of LiOH layer via the newly plated lithium deposits, the subsequent cycles began to deliver a normal voltage range (~ 25 mV). However, the overpotential became extremely unstable after 180 h cycles. This phenomenon indicates that the stability of the Li metal will become worse if LiOH is generated by the water corrosion and covered on its surface. Therefore, the waterproofing of Li metal is necessary during pouch cell assembly. By contrast, the introduction of hydrophobic RI layer on the surface of lithium metal is particularly critical in commercial production. The overpotential of Li symmetric cells is stable even the RI||Li was exposed to dry room for 7 days, confirming its commercialization of practicality. Figure 2d shows the electrochemical impedance spectroscopy (EIS) of Li symmetric cells from 100 kHz to 0.01 Hz before and after the Li was exposed in dry room with the potential of 5 mV. The higher transfer resistance (*R*_ct_) of pristine Li than RI||Li confirms the importance of RI layer on not only suppressing the Li dendrites but also promoting the stability of Li metal even under the risk of water corrosion.

**Fig. 3:**
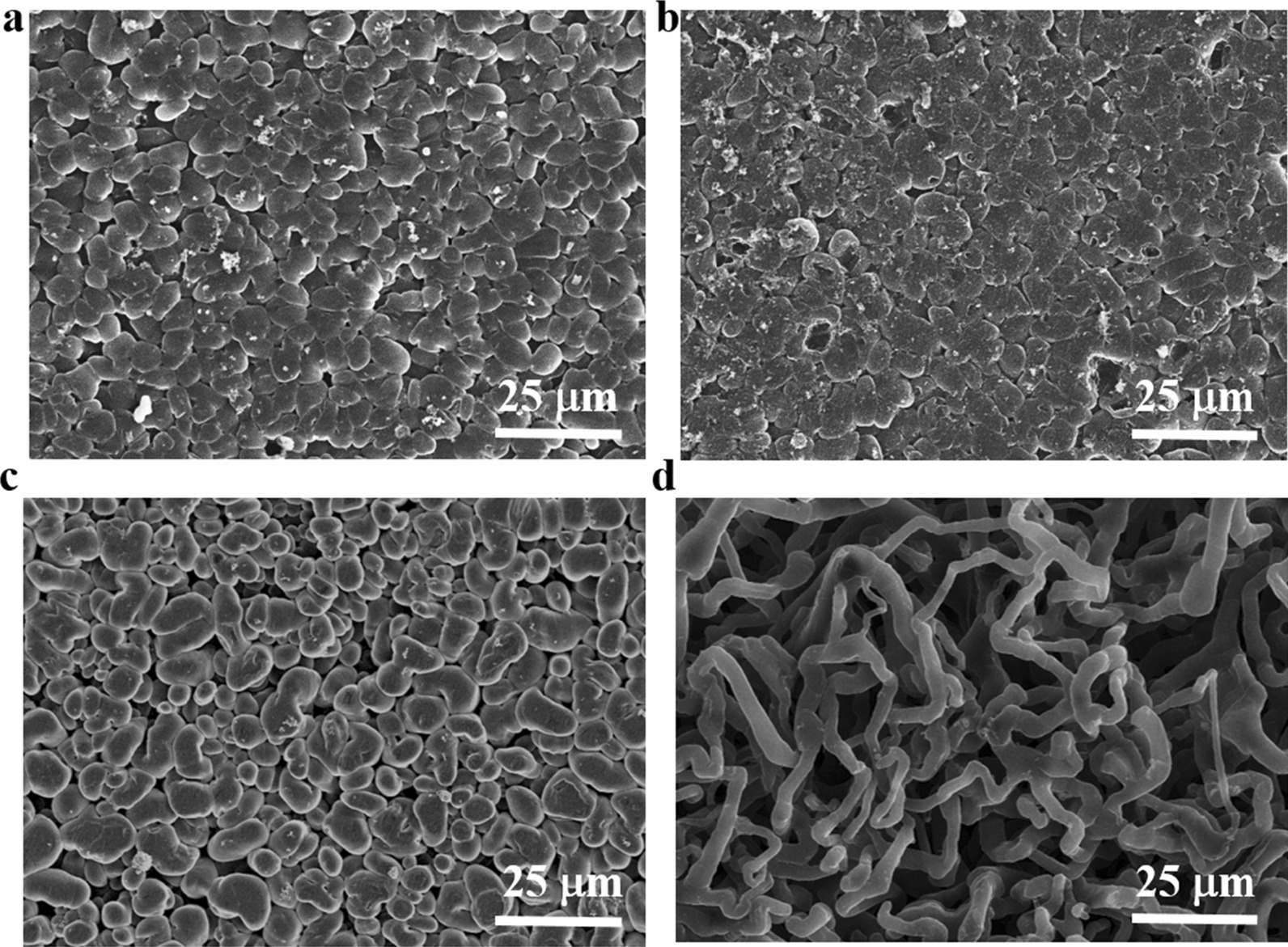
Surface morphologies of the **a** unspoiled RI||Li, **b** corroded RI||Li, **c** unspoiled Li, and **d** corroded Li after the 10th galvanostatic Li stripping/plating cycle with a current density of 1 mA cm^−2^ and a capacity of 1 mAh cm^−2^

Figure 3 shows the surface topography of the cycled Li at a current density of 1 mA cm^−2^ with a capacity of 1 mAh cm^−2^. As shown in Fig. 3a, b, the cycled Li electrode equipped with RI layer possesses dendrite-free Li morphology even being stored in dry room (Fig. 3b). However, on the surface of pristine Li (Fig. 3c, d), plentiful Li dendrite structures appear after Li plating/stripping. After being stored in dry room for 7 days, the cycled pristine Li seemingly leaves the deposited area more disorganized. The Li growth exhibits uncontrolled character accompanied by grievous Li whisker which further confirms the ability of RI||Li anode to stabilize lithium deposition. These results obviously evidence that the as-fabricated RI layer on Li anode can simultaneously isolated water molecules due to its ultra-strong hydrophobic property and suppresses Li dendrite growth due to the generated LiF-rich SEI, which is beneficial for practical application.

### Laboratory-Scale Performance Demonstration of Coin Cells

**Fig. 4:**
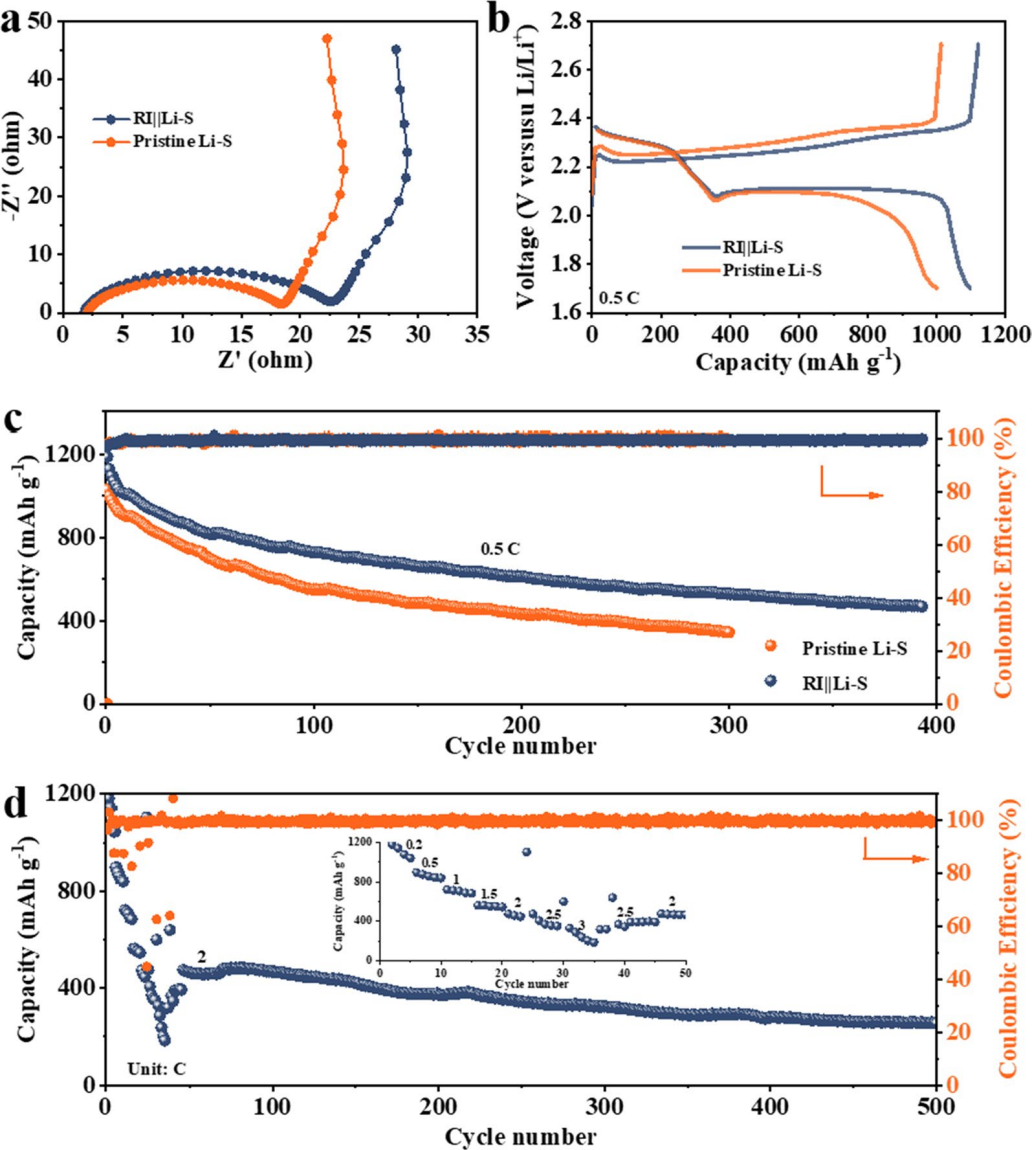
**a** ZK results. **b** Two-plateau charge/discharge profile of the pristine Li and RI||Li at a current rate of 0.5 C. **c** The comparison of cycling performance and Coulombic efficiency with pristine Li and RI||Li at 0.5 C. **d** Rate and long cycle performance of RI||Li

The electrochemistry of Li–S coin cell assembled in argon protected glove box is firstly employed to evaluate the properties of RI||Li. The EIS results show the RI layer causes the increase in charge transfer resistance (*R*_ct_) (Fig. 4a), however, as presented in Fig. 4b, the RI||Li–S cell shows typical voltage curves of Li–S cells, where the location of ~ 2.3 V corresponds to the discharge progress of sulfur to high-order polysulfides. The plateau located at ~ 2.1 V matches the conversion of long-chain polysulfides (2.3 V) to sulfides (2.1 V). The voltage plateau shows that the RI layer does not adversely affect the Li–S cells. The cycle performance of Li–S cells at a low current density of 0.5 C is presented in Fig. 4c. Lower current density for charging/discharging can illustrate the importance of lithium metal anode because once the solubility of polysulfides reaches saturation, the main reason for controlling the cycle stability of the cell will come from the Li metal interface rather than the shuttle effect. Indeed, the shuttle effect of polysulfides dominates the stability of Li–S cells in the initial cycles, allowing only retain ~ 70% capacity retention for both Li–S cells. However, it can be observed that the capacity of RI||Li assembled Li–S cell gradually stabilized with the increase in cycling times (> 100 cycles). This phenomenon further indicates that the inhibition of lithium dendrite can improve the cycle stability. The long cycle performance in Fig. 4d shows that normal redox reaction can take place on RI||Li for more than 500 cycles with capacity retention of ~ 400 mAh g^−1^ at 2 C.

### Electrochemical Performance of Li–S Pouch Cells Assembled with Pristine Li and RI||Li

**Fig. 5:**
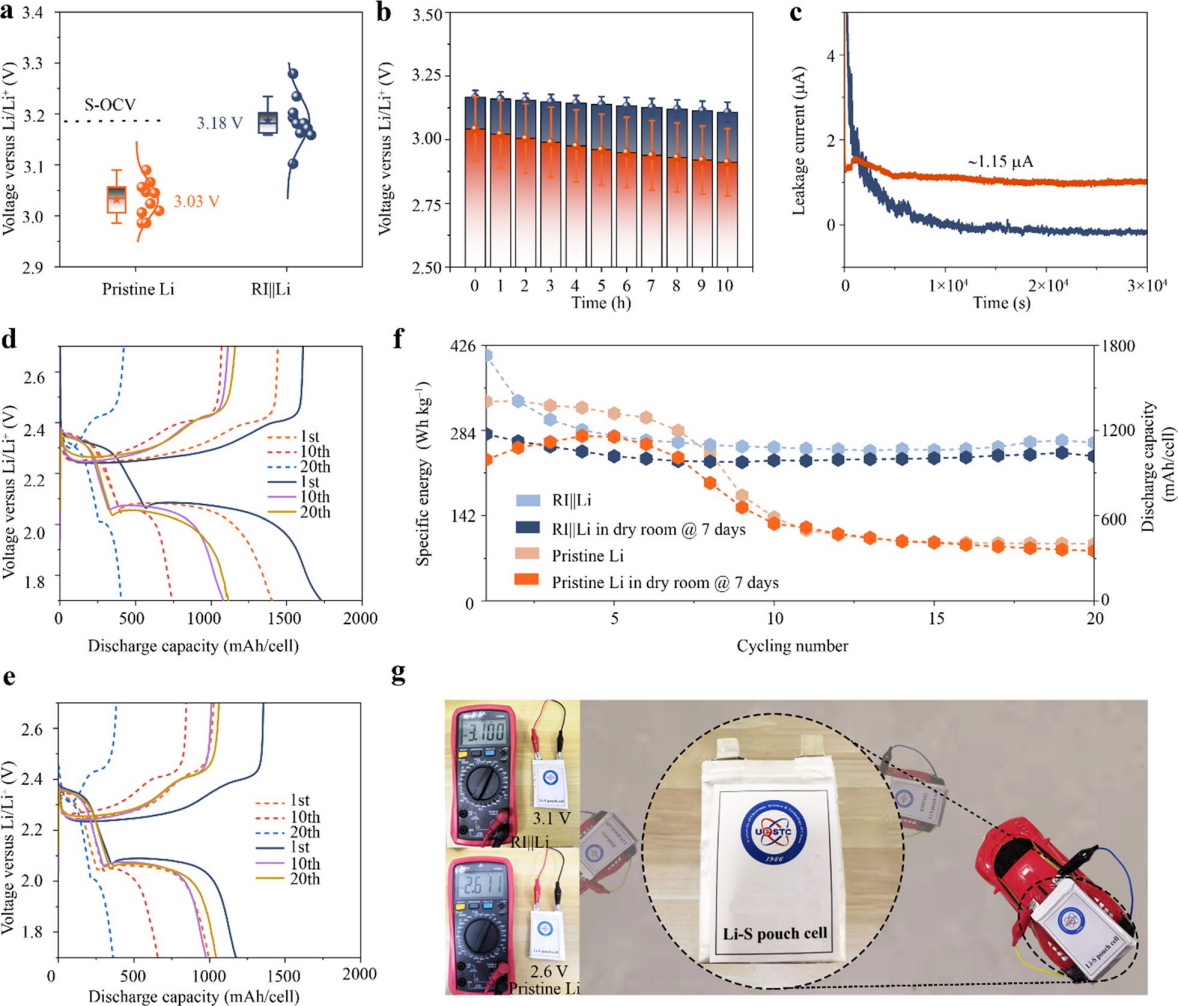
**a** OCV of the Li–S cells coupled with pristine Li or RI||Li. The S-OCV is marked by dashed line. **b** OCV obtained from cells with pristine Li (faint yellow) or RI||Li **(**nattier blue**)** as a function of time. **c** Leakage current of cells with pristine Li (faint yellow) or RI||Li **(**nattier blue**)** during the 3 V constant-voltage floating test. Voltage plateaus of Li–S pouch cells before **d** and after **e** the Li anode was stored in dry room for 7 days and **f** the corresponding cycling performance. The solid curves in voltage profiles represent the Li–S pouch cells with RI||Li, otherwise are pristine Li. **g** Digital photographs of the realistic pouch cell applied in driving the electric car

More in-depth investigation of RI||Li is performed via Li–S pouch cells to confirm the availability. As presented in Fig. 5a, the open circuit voltage (OCV) of Li–S cells with RI||Li anode approaches 3.18 V, which is the theoretical standard OCV (S-OCV) according to the Nernst equation calculated based on 1 M Li ions in electrolyte. The higher OCV than that of Li–S cell assembled with pristine Li (~ 3.03 V) proves that the RI can relieve the formation of passivation layer (internal resistance) via avoiding the spontaneous decomposition of electrolyte on the surface of Li. This is further evidenced by the lower OCV aging of the cell with RI||Li than that with pristine Li (Fig. 5b). To uncover the OCV decline, degradation tests are performed by measuring the leakage current of the cell under a constant voltage of 3 V versus Li^+^/Li. As shown in Fig. 5c, the pristine Li anode presents a continuous leakage current of ~ 1.15 µA during the tests, indicating that the solvent molecules in electrolyte are constantly gaining the electrons from Li anode. In contrast, the leakage current of the cell with RI||Li anode decreases monotonously and reaches 0 μA, which staunchly confirms that the RI-modified Li can endow fewer spontaneous side effects of electrolytes on the surface of Li.

An ampere-hour-scale Li–S pouch cell with ultra-thin Li anode of 50 µm is employed to factually evaluate the practical sustainability of RI||Li under realistic conditions: cathode capacity ≥ 5.0 mAh cm^−2^, *N*/*P* ratio ≤ 2 and *E*/*S* ratio ≤ 4 mL g^−1^. As presented in Fig. 5d, both the Li–S pouch cells with RI||Li and pristine Li show qualified condition to deliver energy densities of more than 300 Wh/kg (counting based on the weight of all cell components, except for the sealants and tabs). However, for the cell with pristine Li, the formation of Li dendrites and the spontaneous decomposition of electrolyte along with the continuous reconstruction of SEI on the surface of Li cause the uninterrupted depletion of electrolyte, leading to the emersion of “dry” positive electrode and SEI. Those make it extremely difficult to release the capacity during the redox reaction. As a consequence, the conversion from long-chain polysulfides to short-chain polysulfides suffers serious depression. The device only releases a capacity of ~ 344.2 mAh/pouch cell (10th cycle). Impressively, 2 times higher capacity (~ 751.7 mAh/pouch cell (10th cycle)) of short-chain polysulfides is obtained when employing RI||Li as anode. This contrast becomes more sharp for the pouch cells assembled with Li anodes after being stored in dry room (Fig. 5e). In addition, pouch cells with RI||Li show better cycle performance with a reversible energy density up to 410 Wh kg^−1^ at a discharging/charging current of 0.2 A/cell (Fig. 5f). The reasons for the improvements can be summarized as the hydrophobic properties of RI layer and the spontaneously formed LiF-rich interphase on Li surface, which guarantees RI||Li with robust SEI structure and dendrites-free Li deposition. Moreover, as shown in Fig. 5g, we successfully demonstrate a realistic application of an ampere-hour-scale Li–S pouch-format cell constructed with 50 µm (single side) RI||Li, which drives the electric car to energetically roam on the floor.

## Conclusions

The pursuit of transforming Li–S coin cells into the commercial pouch cell format is crucial for practicability. In this work, a durable Li metal anode with waterproof performance was created by a hydrophobic RI interface with embedded LiF nanoparticles on the surface of Li. The RI||Li demonstrates high sustainability for achieving an ampere-hour-scale Li–S pouch cell whether or not the storage environment of Li was Ar or dry room (RH = ~ 1.55%). The preparation of RI layer has been shown to be a key way to weaken the bond effects of water molecules on the surface of Li. This approach can be applied to waterproof application of Li metal. Besides, the RI layer, combined with LiF-rich layer, is an ideal backbone to sustainability immobilize Li in actual Li–S pouch cells via decelerating the depletion of electrolyte and the formation of Li dendrites. The Li–S pouch cell reported can supply a high energy density of up to 410 Wh kg^−1^ when employing RI||Li as anode. This work shows the potential of cell technologies from fundamental research to more realistic characterization. Notably, new strategies under realistically large-scale conditions to suppress the shuttle effect of long-chain polysulfides also need to be considered to enable long-term stabilization, especially at the initial state of Li–S pouch cells for future applications.

## Supplementary Information

**Additional file1**. **Figure S1**: Schematic diagram of preparing RI layer by scraping process. **Figure S2**: The contact angels of Li-S electrolyte on Li metal electrode and RI||Li electrode. **Figure S3**: The zoomed-in voltage profiles of Figure 2a. a) 200-300 h. b) 600-700 h.

## Data Availability

All data generated or analyzed during this study are included in this published article.
