# Supplementary material for: Tailoring Lithium Fluoride Interface for Dendrite-Free Lithium Anode to Prolong the Cyclic Stability of Lithium–Sulfur Pouch Cells
Source: Nanoscale Res Lett. 2022 Nov 23;17:112. doi: 10.1186/s11671-022-03745-w (PMC9700540; doi:10.1186/s11671-022-03745-w)
Supplement: Supplementary file 1 — Additional file1. Figure S1: Schematic diagram of preparing RI layer by scraping process. Figure S2: The contact angels of Li-S electrolyte on Li metal electrode and RI||Li electrode. Figure S3: The zoomed-in voltage profiles of Figure 2a. a) 200-300 h. b) 600-700 h. [file 11671_2022_3745_MOESM1_ESM.docx]

**Supporting Information**

**Tailoring Lithium Fluoride Interface for Dendrite-Free Lithium Anode to Prolong the Cyclic Stability of Lithium-Sulfur Pouch Cells**

Li Zhang^1, 2^, Yu Jiao^3^, Fan Wang^1^, Mingjie Zhou^1^, Yin Hu^1^, Yichao Yan^1^, Fei Li^1*^, Tianyu Lei^1^, Bo Chen^4^, Wei Chen^1*^

^1^State Key Laboratory of Electronic Thin Films and Integrated Devices, University of Electronic Science and Technology of China, Chengdu 610054, China.

^2^Tianfu Co-innovation Center, University of Electronic Science and Technology of China, Chengdu 610213, China.

^3^College of Science, Xichang University, Xichang 615000, China.

^4^Institute of Microelectronics of Chinese Academy of Sciences, Beijing 100029, China.

^*^Correspondence: [feili@uestc.edu.cn](mailto:feili@uestc.edu.cn); [weichen@uestc.edu.cn](mailto:weichen@uestc.edu.cn)

Full list of author information is available at the end of the article.


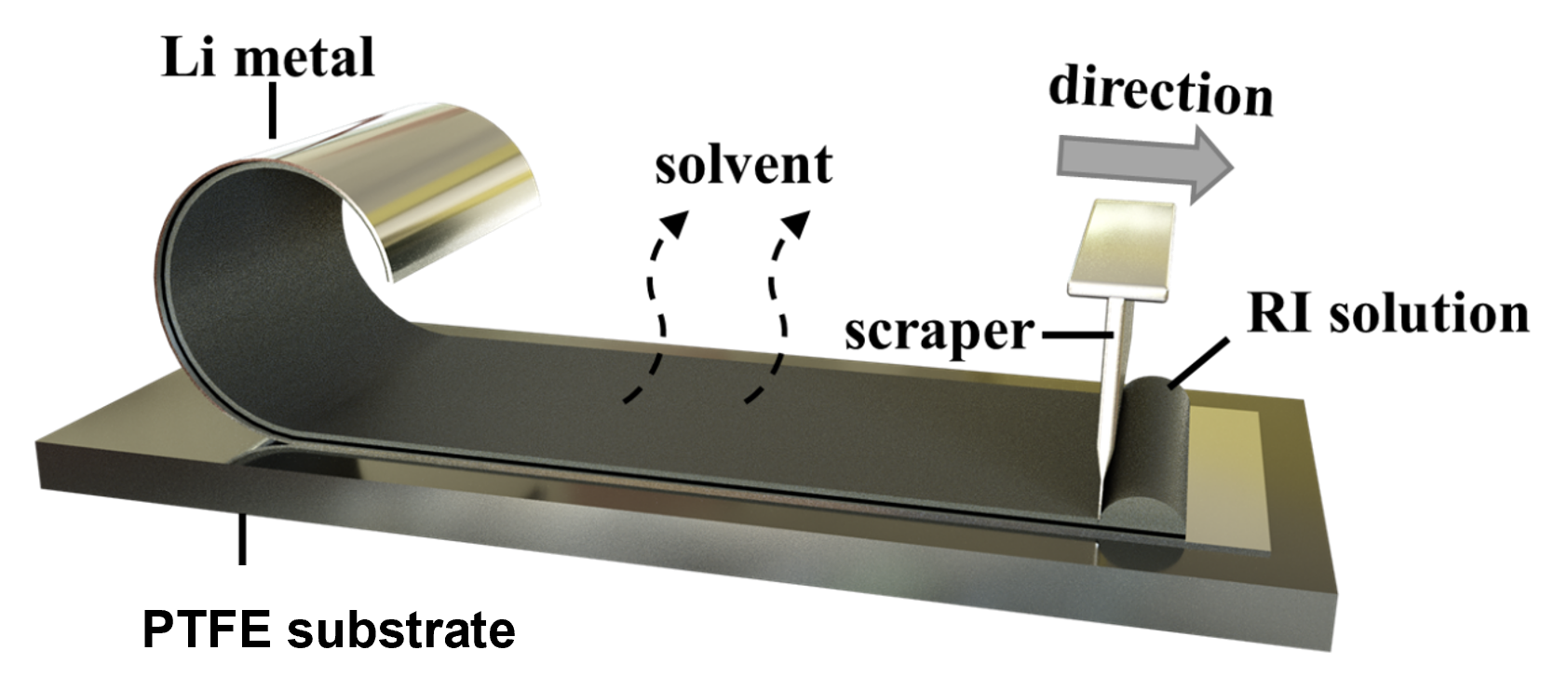


Figure S1. Schematic diagram of preparing RI layer by scraping process.


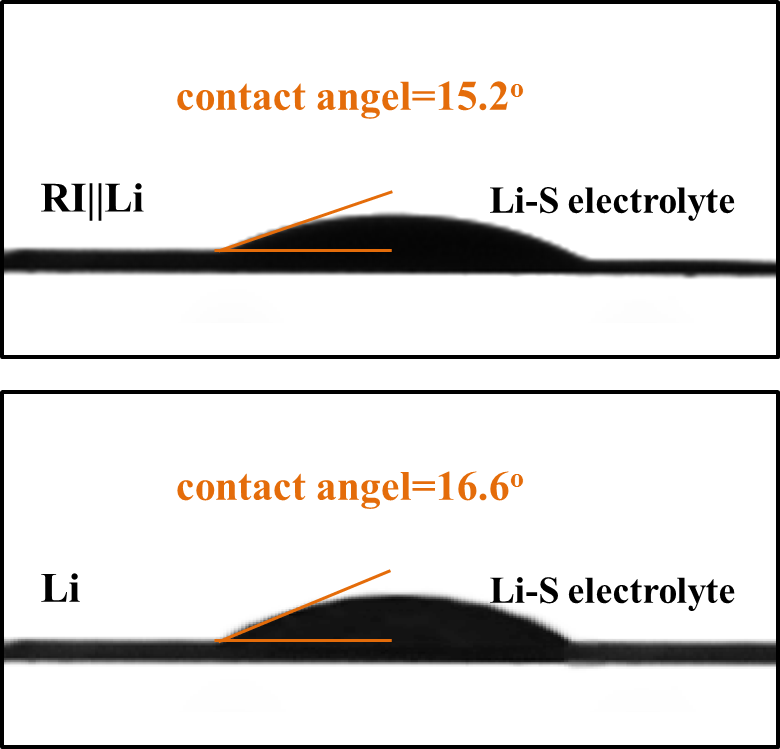


**Figure S2.** The contact angels of Li-S electrolyte on Li metal electrode and RI||Li electrode.


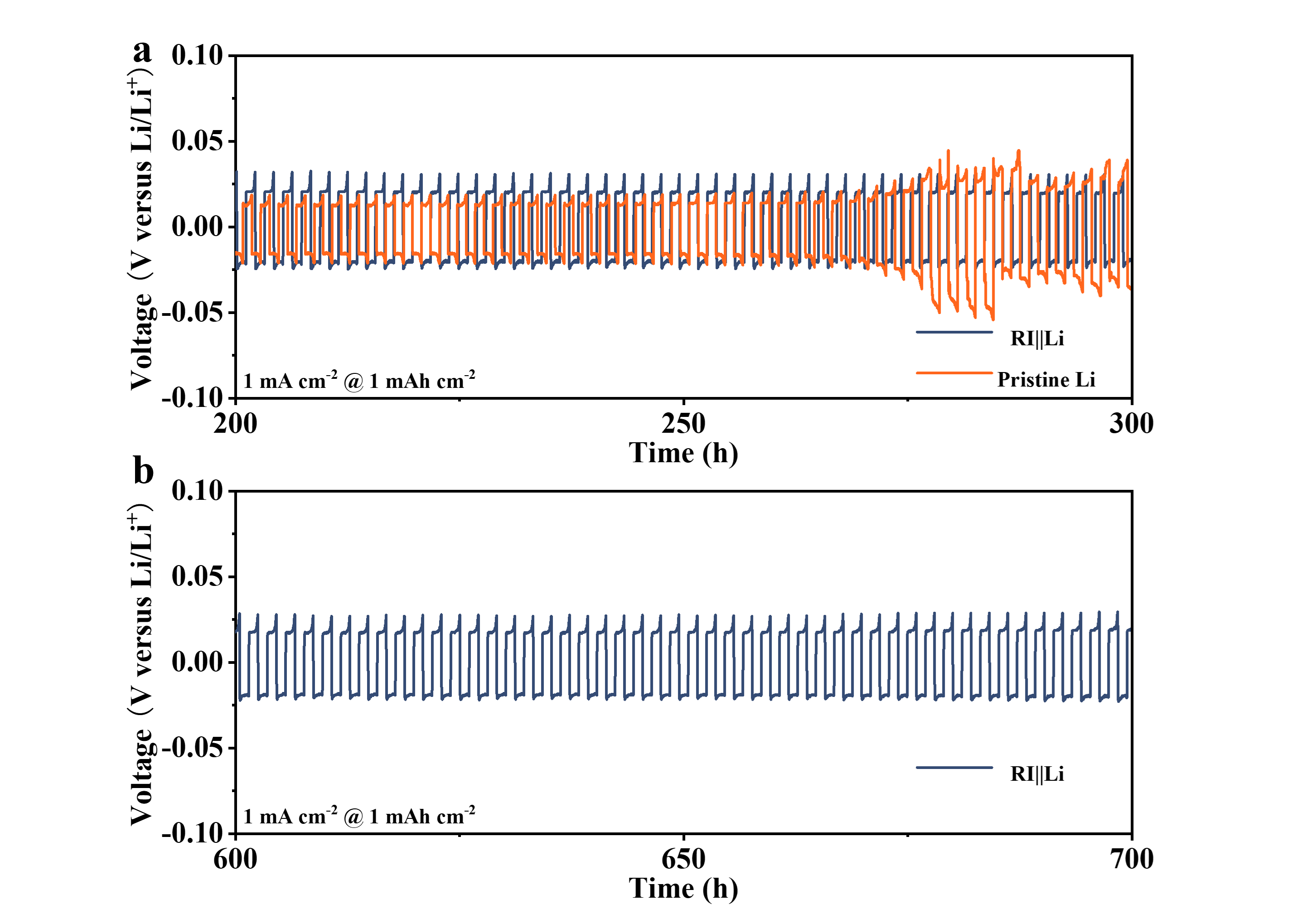


**Figure S3.** The zoomed-in voltage profiles of Figure 2a. a) 200-300 h. b) 600-700 h.
